# Using artificial intelligence on dermatology conditions in Uganda: a case for diversity in training data sets for machine learning

**DOI:** 10.4314/ahs.v23i2.86

**Published:** 2023-06

**Authors:** Louis Kamulegeya, John Bwanika, Mark Okello, Davis Rusoke, Faith Nassiwa, William Lubega, Davis Musinguzi, Alexander Börve

**Affiliations:** 1 The Medical Concierge Group, Research and Projects; 2 The Medical Concierge Group, Information and Technology; 3 Sahlgrenska University Hospital, Departments of Orthopaedics

**Keywords:** Artificial intelligence, Dermatology, Fitzpatrick 6 skin type, Telehealth, Algorithms

## Abstract

**Background:**

In pursuit of applying universal non-biased Artificial Intelligence (AI) in healthcare, it is essential that data from different geographies are represented.

**Objective:**

To assess the diagnostic performance of an AI-powered dermatological algorithm called Skin Image Search on Fitzpatrick 6 skin type (dark skin) dermatological conditions.

**Methods:**

123 dermatological images selected from a total of 173 images were retrospectively extracted from the electronic database of a Ugandan telehealth company, The Medical Concierge Group (TMCG) after getting their consent. Details of age, gender, and dermatological clinical diagnosis were analysed using R on R studio software to assess the diagnostic accuracy of the AI app along with disease diagnosis and body part. Predictability levels of the AI app were graded on a scale of 0 to 5, where 0- no prediction was made and 1-5 demonstrated a reduction incorrect diagnosis prediction rate of the AI.

**Results:**

76 (62%) of the dermatological images were from females and 47 (38%) from males. Overall diagnostic accuracy of the AI app on black dermatological conditions was low at 17% (21 out of 123 predictable images) compared to 69.9% performance on Caucasian skin type as reported from the training results. There were varying predictability levels correctness i.e., 1-8.9%, 2-2.4%, 3-2.4%, 4-1.6%, 5-1.6% with performance along individual diagnosis highest with dermatitis (80%).

**Conclusion:**

There is need for diversity of image datasets used to train dermatology algorithms for AI applications to increase accuracy across skin types and geographies.

## Introduction

Dermatological conditions are among the leading cause of non-fatal disease burden globally yet receive little attention in terms of prevention, policy, and clinical management 1,2. When comparing absolute years lived with disability (YLDs), the global burden of disease study reported skin diseases as the fourth leading cause of disability, after iron-deficiency anemia, tuberculosis, and sense organ diseases 3. The prevalence rates of skin diseases take on a geographical and socio-economic variation with limited resources in settings like sub-Saharan Africa, having a high prevalence of dermatological conditions like viral warts, pyoderma, cellulitis, scabies, psoriasis, alopecia areata, urticaria, fungal skin diseases, and decubitus ulcers 4.

Little research has been documented on the prevalence and burden of skin diseases in Uganda. The few existing studies show endemic patterns of dermatological conditions in some regions of the country, like the Visceral Leishmaniasis in the north-eastern districts of Moroto and Kotido and Buruli ulcer in the western and north-western parts of the country 5. Studies have highlighted the socioeconomic impact of dermatological illnesses on affected subpopulations including reduced productivity from disease state and cost of treatment 6.

A number of factors contribute to this observed trend in the occurrence of skin diseases in Uganda and these include living conditions that bring humans closer to the vector e.g., brick and mud huts, close proximity to water bodies, poor disposal of human waste, and sharing homesteads with animals among others. These conditions provide a favourable environment for the proliferation of skin diseases like skin fungus and embryonic blood flukes that nestle into their human hosts' 7. These situations are further boosted by water insecurity in the communities coupled with the lack of proper treatment support systems in our limited resource settings 8.

In a country where the doctor-to-patient ratio approximates 1:25,000 and worse for specialists like dermatologists, access to specialized medical services like dermatology is a very challenging 9. Therefore, it is not uncommon for health conditions that require a specialist opinion to go undiagnosed causing personal social issues with associated morbidity. Innovations like the use of AI aided platforms as a diagnostic tool to assist in diagnosing and choosing the correct treatment plan on the first visit could help to bridge the gap of low numbers of dermatologists in limited-resource settings.

AI is defined as “the ability of a computer to mimic intellectual processes characteristic of humans, such as the ability to reason, discover meaning, generalize, or learn from past experience in order to achieve goals without being explicitly programmed for specification.” 10. The use of AI has already shown immense potential with its integration in areas including; education, linguistics, agriculture and among others 11–13. This has not left the health sector with many AI based innovations successfully being applied for image analysis in radiology, anesthesiology and pathology with benefits including reduction on medical care cost while providing quicker diagnosis 14,15.

Concerns about the diagnostic accuracy of deep learning computer vision aided tools in healthcare have been noted, a study analysing the performance of an imaging analysis computer algorithm showed tendencies of over diagnosis 16. A number of factors have been attributed to this suboptimal performance of computer vision tools in health and among them is the lack of diversity in datasets used for training the algorithms which often excludes certain demographics especially blacks 17–19.

In the majority of scenarios, these AI-based solutions are designed from the western and European world with their algorithms trained in western/European world environments. This by default leaves out specific demographics who in most cases these solutions are being designed to solve their problems, a fallacy! It is important to note that machine learning algorithms sift through millions of training data points to make correlations and predictions, as such developing models that embrace people from different backgrounds and communities is critical to having an all-inclusive AI experience. In view of this, we carried out a study to assess the diagnostic accuracy of an AI algorithm developed by First Derm using a deep convolutional neural network (CNN) called “Skin Image Search” 20 on Fitzpatrick 6 skin types (black-dark) dermatological conditions.

## Methods

### Study Design

An observational study with descriptive statistical analysis of independent datasets containing Fitzpatrick 6 skin type dermatological conditions that were retrospectively extracted from the Telehealth platforms of a digital health company called The Medical Concierge Group (TMCG), located in Kampala-Uganda 21.

### Data Collection

TMCG operates a 24/7 digital health platform manned by qualified and licensed doctors that provide remote resolution to users' medical inquiries. Dermatological inquiries involve users sharing images of the skin condition via the tele-platforms using their smartphones with additional history taking by the clinician to assess associated factors including onset, pattern, exacerbating or relieving factors, and chronicity among others as means to reach the correct diagnosis. Each individual image was reviewed by 3 independent general practitioners for which the final clinical diagnosis of the image was the most reported diagnosis. There was no scenario where the final diagnosis for an image differed among all the 3 practitioners.

In instances where there was a disagreement, the final diagnosis was achieved by consensus. The shared images were anonymized prior to storage in the electronic medical records database. In this study, dermatological images between January to March 2018 were selected according to inclusion and exclusion criteria (see below). Data collection was done by consecutive sampling, where images were collected until the desired sample size i.e., dermatological images shared from January to March 2018 was acquired. All included images were imported by their file names into Microsoft Excel 2019 22 and the following additional information was added; patient ID, gender, body site and final diagnosis (defined as the clinical diagnosis).

### Inclusion Criteria

All consecutive dermatological images within the TMCG tele-platform from January to March 2018 were included. All images were anonymous since they contained no identifying data, and the patient could not be recognized by the image. When necessary, this was achieved by cropping the image in Microsoft paint 22. See the appendix on how to crop images in Microsoft Paint.

### Exclusion Criteria

Nonassessable images due to image quality, (for example bad lighting or blurred focus) or unfit image composition (camera not aimed at skin lesion, image taken from inadequate distance or angle) were excluded. Images showing pen markings like circles or arrows were excluded as well as where the skin lesions were wholly or partially covered by a dressing e.g., bandage. Images with more than one diagnosis were cropped in a way that the confirmed diagnosis became the main visible lesion in the image. For example, images that could have a burn and a fungal skin lesion.

Skin Image Search™ – an artificial intelligence Dermatology and Venereology classification system First Derm is a store and forward tele dermatology service that delivers advice for users with skin conditions by a team of international board-certified dermatologists based on two uploaded smartphone images and associated information in the text. First Derm gives easy access to a dermatologist for advice, which otherwise can be time-consuming and more expensive for patients. According to the company, about 80 % of their users are helped by over-the-counter medication. 20 % require an additional appointment for further testing and additional treatments.

Over a 10-year period First Derm has received hundreds of thousands of amateur smartphone images from tele-consultations portraying a large number of skin conditions. Using a data set of 63,237 images (Inflammatory 44.5k images, Tumor 11k images, Genitalia 4,571 images and 3,166 other skin disease images. Where it is estimated that 5-10% were of black skin texture. With these images, an AI-algorithm evolved by training a CNN and developed their first classification system version 33.1, with ability to identify 33 diseases in Dermatology and Venereology. This algorithm called Skin Image Search™ (from here on, the AI app) is a free and fast service, available online.

The user uploads two images from their smartphone showing their skin condition, and the online service searches its dataset for matching images and within seconds, returns a list with the top 5 most likely diagnoses, in descending order from 1 to 5, hereby referred to as the “top 5”. The reasons to upload two pictures is to achieve confirmation of exact dermatological condition being diagnosed through comparison and the AI will analyse the best-fit image of the two uploaded pictures. The second picture offers a ‘second eye’ to the AI and helps in comparison and confirmation purposes with the first one to avoid any inconsistencies that would result in missed or misdiagnosis.

According to the company, the AI app has shown results of 31.6% accuracy in returning the true diagnosis ranked as the most likely. The accuracy in the top 5, e.g., the presence of the true diagnosis among the five proposed differential diagnoses, was 69.9%. The company is continuously experimenting with its CNN and hope to reach higher diagnostic accuracy in future versions with coming updates of the AI app.

### Testing the AI classification system

**Figure 1:**
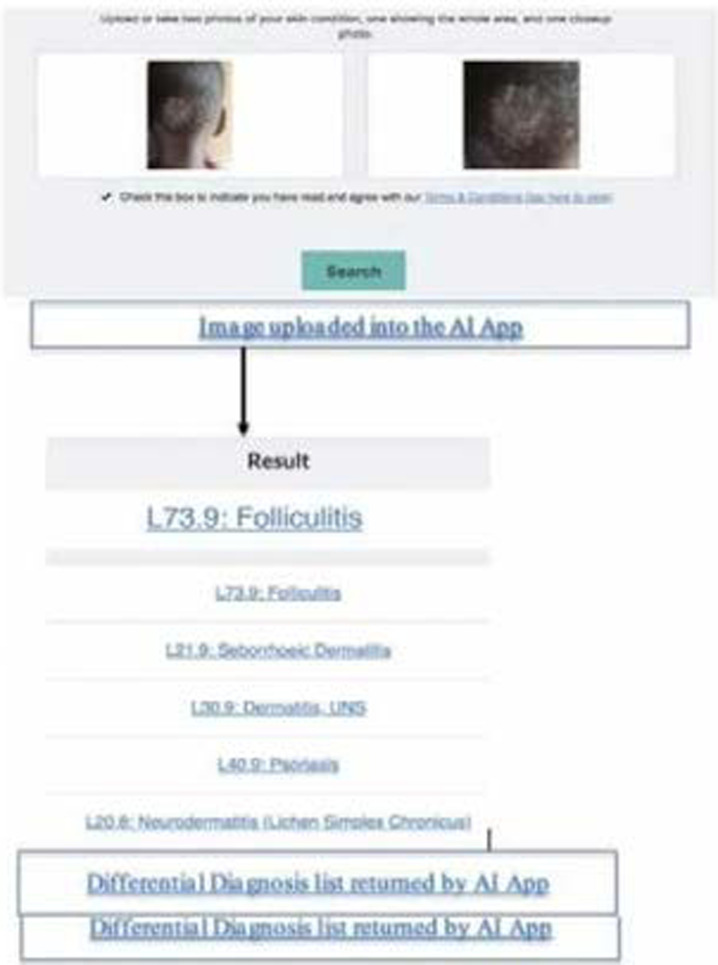
Illustration of the testing process of the AI app

Collected images were uploaded for automated classification using an online version of the AI app 20 which required uploading two images, one showing the wider body area where the lesion is situated and second closeup photo to allow the classification process to be made as illustrated in figure 1. The top 5 differential diagnoses returned from the AI app against each individual image that was tested were imported into Microsoft Excel sheet. Matching the image's clinical diagnosis with the returned top 5, each classification was given a score from 1-5 depending on which position the confirmed diagnosis had, score 1 being the most likely. If the confirmed diagnosis was absent in the top 5, the classification was given a score of 0.

### Diagnostic accuracy

The overall diagnostic accuracy of the AI app was analysed as well as the diagnostic accuracy for separate diagnoses, diagnostic groups with a common etiology (e.g., tumors, viral diseases, and fungal diseases), or body site (e.g., genital diseases and facial diseases). The dermatological diagnosis analysed by the AI app including the relevant diagnostic groups is presented in Table 1.

**Table 1:** Presentation of the diagnoses with respective diagnostic codes (ICD-10) for dermatological conditions tested with the AI app in this study

Diagnostic group- N (%)	Diseases diagnoses	ICD-10
**Bleeding disorders**		
2 (1.6%)	Ecchymoses	R233
**Burns**		
4 (3.2%)	Burns	L550
**Chickenpox**		
4 (3.2%)	Varicella	B01
**Dermatitis**	Impetigo	L01
20 (16.2%)	Eczema	B000
	Psoriasis	L40
**Facial diseases**		
9 (7.3%)	Acne Vulgaris	L70
**Folliculitis**		
4 (3.2%)	Folliculitis	L662
**Fungal Diseases**	Tinea cruris	B356
33 (26.8%)	Tinea corporis	B354

### Statistical analyses

All data was wrangled and analysed using R version 3.6.0 on R Studio Version 1.2.1335. The data analysis plan was drafted and followed, the data was majorly categorical (92%) and numerical (8%). All categorical variables were nominal (i.e., sex and body part) apart from the target which was ordinal (dermatological diagnosis). The data was cleaned and wrangled using dplyr package and exploratory data analysis using ggplot2 and vcd packages for univariate and multivariate respectively performed on cleaned data and insights derived and communicated.

### Ethics

Institutional approval for usage of the images within TMCG's electronic medical records was granted. Image file names did not include any patient data and the patients' identity could not be discerned in the images used in this study. Furthermore, the use of images in this study did not affect patients' healthcare.

## Results

A total of 173 dermatological images were gathered from TMCG's electronic medical records. However, 50 images were excluded according to the exclusion criteria above, resulting in a final number of 123 images included and uploaded one by one for classification in the AI app. The gender divide of the study population was 47 males (38 %) and 76 females (62%) with a median age of 23 years. See Table 2 for the demographic characteristics of the population.

**Table 2:** Demographic characteristics

Population size N= 123	
**Age (years)**	
Minimum	0.04
Maximum	75
Median	23
Mean	19.37
25th percentile	4
75th percentile	28
**Gender**	
Female	76(62%)
Male	47 (38%)
**Skin Type**	
VI	123 (100%)

The five most common body sites were genital (20%), trunk (20%), lower limb (14.6%), face (12%) and upper limb (12%). See Table 3 below.

**Table 3:** Image count about body site

Body Site	Image count (%)
Anal	2 (1.6%)
Trunk	1 (0.8%)
Ear	1 (0.8%))
Face	15 (12%)
Genitals	24 (20%)
Lips	1 (0.8%)
Lower limb	18 (14.6%)
Lower limb Extremities	5 (4%)
Neck	6 (4.9%)
Oral Cavity	6 (4.9)
Scalp	5 (4%)
Trunk	24 (20%)
Upper limb	15 (12%)

The diagnostic accuracy of the AI app is presented initially with general results i.e., overall disease diagnostic accuracy, and thereafter with more specific results i.e., accuracy about other variables i.e., body size and gender.

### Disease diagnostic accuracy of AI

**Figure 2:**
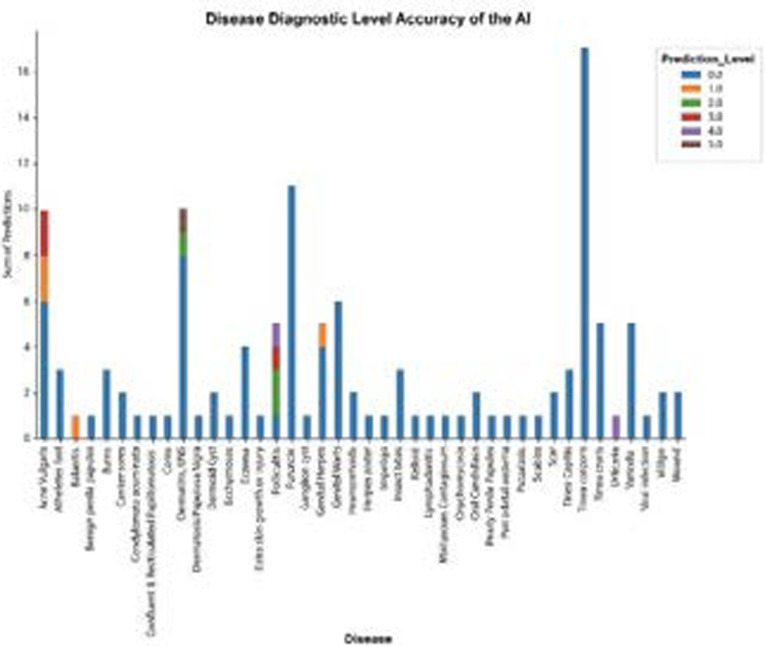
Overall disease diagnostic accuracy of the AI app

Out of the 123 images uploaded for classification, the AI app was able to place the correct diagnosis among the top 5 in 17% of the images (21/123) and failed to return the correct diagnosis in 83% of the images (102/123). The varying predictability level correctness of the AI app was 1-8.9%, 2- 2.4%, 3- 2.4%, 4- 1.6%, and 5- 1.6%, making the true diagnosis (level 1) the largest portion of the other scores in the top 5 (Figure 2). The AI app performed very well on Dermatitis with 80% of uploaded images being predicted with the correct diagnosis (level 1). The AI app performed poorly on Tinea (capitis, corporis, and cruris) images yet these had the biggest count.

### Body part diagnostic level accuracy of the AI app

**Figure 3:**
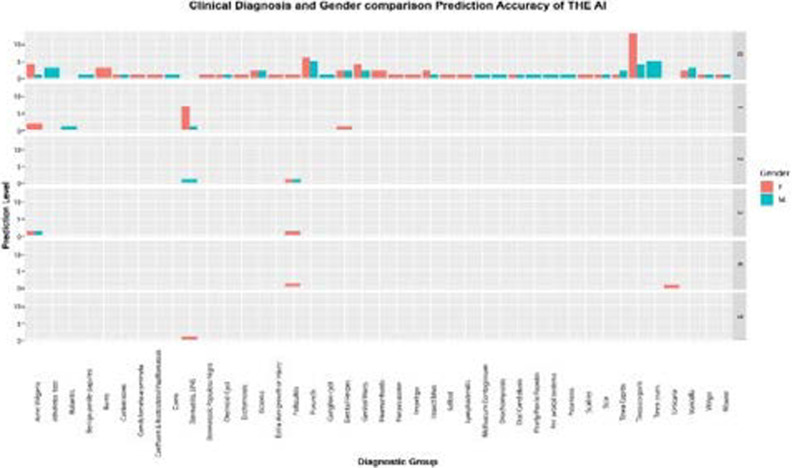
Diagnostic accuracy of the AI app by a body part of the dermatological image

Out of the 123 images uploaded; the AI app returned a diagnosis in 62% of all body parts (8/13). The AI app performed well in dermatological images from the face, trunk, and genital areas and lowest for lower limb images (Figure 3).

### Clinical diagnosis and gender comparison prediction of the AI app

**Figure 4:**
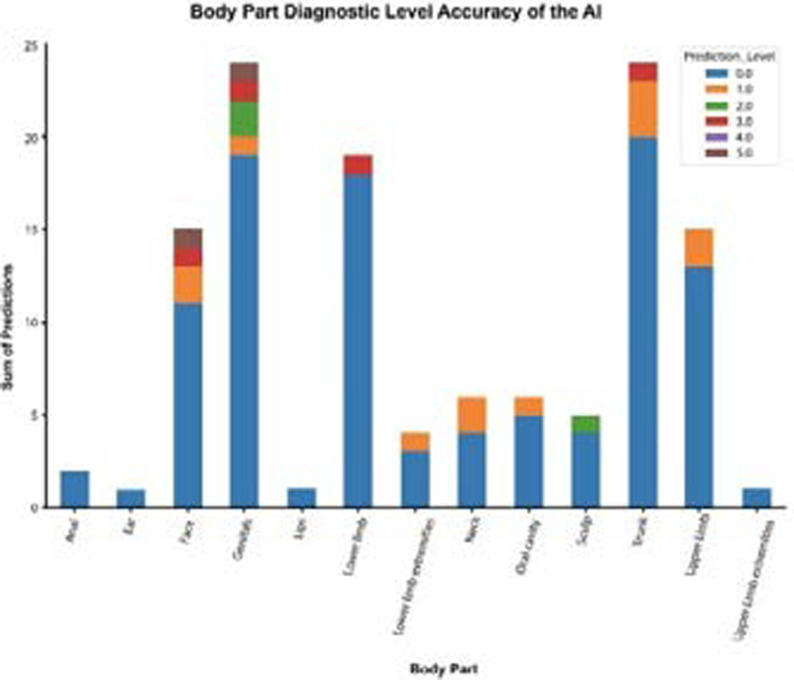
Gender comparison of the diagnostic accuracy of the AI app across dermatological clinical diagnoses

Overall, the AI app performed slightly better among females compared to males for the same dermatological diagnosis. For example, for Dermatitis, the overall performance in females was 70% compared to 20% in males, and the same pattern is noted for Acne Vulgaris (F- 33%, M-11%) and folliculitis (F-60 %, M-20 %). (See figure 4).

### Body part and gender comparison prediction of the AI app

**Figure 5:**
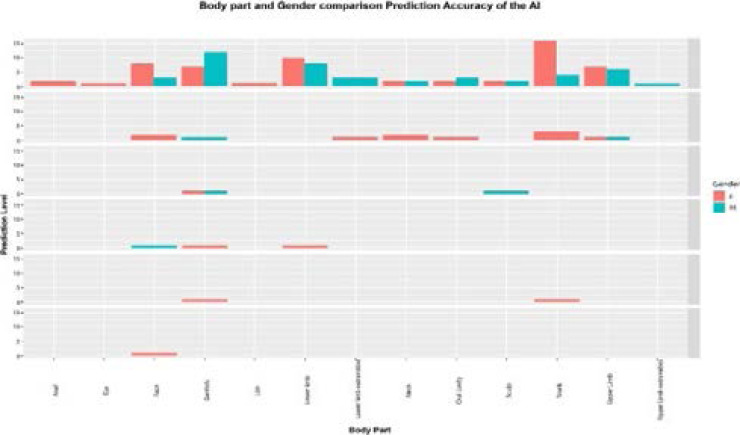
Gender comparison of the diagnostic accuracy of the AI app along with body parts of the dermatological images

AI app predictability of correct diagnosis along body parts showed some trends of gender preference with better performance noted in females compared to males. For example, for facial images, the AI app performance among females was 20% compared to 6.7% in males; for genital images, performance among females was 12.4% compared to 8.3% in males; for images of the trunk, performance among females was 8.3% compared to 0% in males, etc. (See figure 5).

## Discussion

First-Derm was an AI-powered dermatology application under development and TMCG a digital health company was chosen to provide an external beta-testing in a black African environment. This manuscript discusses the learning from testing First-Derm with Fitzpatrick 6 skin types (African black skin) dermatological conditions. Fungal diseases accounted for the majority of dermatological images reviewed (26.8%), these findings relate to the 2010 global burden of disease report which placed fungal diseases as the most prevalent among the top 10 skin diseases globally. Environmental factors including the warm humid weather have been noted to contribute to an increased risk of fungal infestations which are very consistent with the Uganda geographical setting 23.

The study population was relatively young with a median age of 23 years, this is largely from the fact that these images were from the electronic medical records of a telehealth platform for which studies have always related young age with uptake and usage of digital platforms for medical care 24,25. This further creates an opportunity to leverage AI applications for dermatological medical care among the young people who often do not have enough resources and rely on peer-to-peer consultations when managing skin problems, a trend seen with other medical problems like mental, sexual, and reproductive health 26.

The overall diagnostic accuracy of the AI app was slightly poor at 17% (21/123) compared to the 69.9% performance reported from the AI app training results. This is an indicator that the First-Derm application was mainly trained based on loads of Fitzpatrick 1&2 skin types (Caucasian skin) and fewer of Fitzpatrick 5 & 6 skin types (Dark-coloured skin) who make up the majority of images that were used for this testing. This finding correlates with other studies on the potential racial bias of AI and machine learning algorithms 27,28.

Performance of the AI app along specific dermatological skin diagnosis showed interesting results with up to 80% correct diagnosis predicted for Dermatitis and no return (0%) for fungal diseases, yet these had the highest count. These findings imply that the AI app was trained adequately on dermatitis datasets compared to fungal images which relate to the fact that the prevalence of dermatitis in western and European settings is high compared to the fungal infestations 2,29.

In addition, the AI app performed poorly on tumor diagnostic groups (i.e., scar tissue, dermoid cyst, corn, ganglion cyst). This could partly be explained from the angle that the application had not yet been trained with several anatomical structures to delineate the true body structure from which the skin condition has been taken. However, the AI app returned a diagnosis in 62% of all body parts (8/13) an indicator that the AI app had been trained with images from a variety of different body sites.

Comparison of the AI app diagnostic accuracy along gender for individual dermatological diagnosis and body parts showed a slightly better performance in females compared to males. It is not clear as to what may explain this observation, however, viewing it from the angle of skin tone and texture is smoother among African women than men may explain this observation. The First Derm application like any other AI app is not able to integrate the client's history to a presenting dermatology inquiry in making its list of differentials, as such instances where history to the occurrence of complaints is critical in making the diagnosis for example in cases of burns and wounds such technology-based diagnostic tools are likely to make a miss-diagnosis. This is further echoed by the notion that if an AI program is not trained to perform a particular task it will not be able to execute its 10.

## Conclusions

AI is well suited for the classification of skin disease, for any classification to be universal; there is a need for diversity in the images used when training CNNs. Diversity in dermatological image datasets will help reduce biases, and the need to include many different kinds of skin complexities; Caucasian, dark-coloured, brown, and African-black skin colours will help achieve significantly better diagnostic accuracy results.

## References

[1] Darmstadt. Institute for Health Metrics and Evaluation (2018). Findings from the Global Burden of Disease Study 2017. The Lancet.

[2] Mahé A, Hay JR, Carapetis J, Darmastadt G, MacLennan C, Melis de la Vega M (2005). Epidemiology and Management of Common Skin Diseases in Children in Developing Countries. World Health Organ Dep Child Adolesc Health Dev.

[3] Seth D, Cheldize K, Brown D, Freeman EF (2017). Global Burden of Skin Disease: Inequities and Innovations. Curr Dermatol Rep.

[4] Hay RJ, Johns NE, Williams HC, Bolliger IW, Dellavalle RP, Margolis DJ (2014). The Global Burden of Skin Disease in 2010: An Analysis of the Prevalence and Impact of Skin Conditions. J Investig Dermatol Home.

[5] Olobo-Okao J, Sagaki P (2014). Leishmaniasis in Uganda: Historical account and a review of the literature. Pan Afr Med J.

[6] Kolaczinski JH, Kabatereine NB, Onapa AW, Ndyomugenyi R, Kakembo ASL, Brooker S (2007). Neglected tropical diseases in Uganda: the prospect and challenge of integrated control. Trends Parasitol.

[7] Loewenberg S (2014). Uganda's struggle with schistosomiasis. The Lancet[Internet].

[8] Ovuga EB, Okello DO, Ogwal-Okeng JW, Orwotho N, Opoka RO (1995). Social and Psychological Aspects of Onchocercal Skin Disease in Nebbi District, Uganda. East Afr Med J.

[9] Matsiko CW, Kiwanuka J (2003). A Review of Human Resource for Health in Uganda. Health Policy Dev.

[10] Bali J, Garg R, Bali RT (2019). Artificial intelligence (AI) in healthcare and biomedical research: Why a strong computational/AI bioethics framework is required?. Indian J Ophthalmol.

[11] Timms MJ (2016). Letting Artificial Intelligence in Education Out of the Box: Educational Cobots and Smart Classrooms. Int J Artif Intell Educ.

[12] Fu X, Lu W, Zhu L, Zhou S (2017). Study of the Establishment of a Reliable English-Chinese Machine Translation System Based on Artificial Intelligence. Lecture Notes in Real-Time Intelligent Systems [Internet].

[13] Jha K, Doshi A, Patel P, Shah M (2019). A comprehensive review on automation in agriculture using artificial intelligence. Artif Intell Agric.

[14] Mathis MR, Kheterpal S, Najarian K (2018). Artificial Intelligence for Anesthesia: What the Practicing Clinician Needs to Know. Anesthesiology.

[15] Chang HY, Jung CK, Woo IJ, Lee S, Cho J, Kim SW (2019). Artificial Intelligence in Pathology. J Pathol Transl Med.

[16] Perrinaud A, Gaide O, French LE, Saurat JH, Marghoob AA, Braun RP (2007). Can Automated Dermoscopy Image Analysis Instruments Provide Added Benefit for the Dermatologist? A Study Comparing the Results of Three Systems. Br J Dermatol.

[17] Kwon O, Sim JM (2013). Effects of data set features on the performances of classification algorithms. Expert Syst Appl.

[18] Gianfrancesco MA, Tamang S, Yazdany J, Schmajuk G (2018). Potential Biases in Machine Learning Algorithms Using Electronic Health Record Data. J Am Med Assoc Intern Med.

[19] Marchetti MA, Codella NCF, Dusza SW, Gutman DA, Helba B, Kalloo A (2018). Results of the 2016 International Skin Imaging Collaboration International Symposium on Biomedical Imaging Challenge: Comparison of the Accuracy of Computer Algorithms to Dermatologists for the Diagnosis of Melanoma from Dermoscopic Images. J Am Acad Dermatol.

[20] (2020). First Derm. Skin Image Search [Internet].

[21] Kamulegeya L, Ssebwana J, Abigaba W, Bwanika J, Musinguzi D (2019). Mobile Health in Uganda: A Case Study of the Medical Concierge Group. East Afr Health Sci [Internet].

[22] Microsoft (2020). Microsoft Corporation [Internet].

[23] Talley SM, Coley PD, Kursar T (2002). The effects of weather on fungal abundance and richness among 25 communities in the Intermountain West. BMC Ecol [Internet].

[24] Buhi ER, Klinkenberger N, Hughes S, Blunt HD, Rietmeijer C (2013). Teens' Use of Digital Technologies and Preferences for Receiving STD Prevention and Sexual Health Promotion Messages: Implications for the Next Generation of Intervention Initiatives. Sex Transm Dis.

[25] Selkie EM, Benson M, Moreno M (2011). Adolescents' Views Regarding Uses of Social Networking Websites and Text Messaging for Adolescent Sexual Health Education. Am J Health Educ.

[26] Martínez-Hernáez A, DiGiacomo SM, Carceller-Maicas N, Correa-Urquiza M, Martorell-Poveda MA (2014). Non-professional-help-seeking among young people with depression: a qualitative study. BMC Psychiatry [Internet].

[27] Howard A, Borenstein J (2018). The Ugly Truth About Ourselves and Our Robot Creations: The Problem of Bias and Social Inequity. Sci Eng Ethics.

[28] Noor P (2020). Can We Trust AI Not to Further Embed Racial Bias and Prejudice?. Br Med J [Internet].

[29] Kowalska-Olędzka E, Czarnecka M, Baran A (2019). Epidemiology of atopic dermatitis in Europe. J Drug Assess.

